# Changes in social inequality with respect to health-related living conditions of 6-year-old children in East Germany after re-unification

**DOI:** 10.1186/1471-2458-5-64

**Published:** 2005-06-08

**Authors:** Xianming du Prel, Ursula Krämer, Ulrich Ranft

**Affiliations:** 1 Institut für Umweltmedizinische Forschung (IUF) Heinrich-Heine-University Duesseldorf, Auf'm Hennekamp 50, D-40225 Duesseldorf, Germany

## Abstract

**Background:**

Since Germany re-unified in 1990, substantial social and economic changes have happened in East Germany, the former socialist German Democratic Republic (GDR). The aim of this study was to investigate the influence of these socio-economic changes in East Germany on the association between social status, measured by parental educational level, and health-related living conditions of children during the ten-year period after re-unification.

**Methods:**

In total, 25,864 6-year-old school beginner children (51.2% male and 48.8% female) participated in cross-sectional studies which have been repeated every year from 1991 to 2000 in East Germany. Parental educational level as a social indicator was the independent variable. Dependent variables included not employed parents, small living space and health-related living conditions (e. g. damp housing, single oven heating and living at busy road). The relationships were described by odds ratios using logistic regression.

**Results:**

A large overall effect of parental educational level on health-related living conditions was observed. The time trends showed that the situation regarding small living space, damp housing conditions and single oven heating improved from 1991 to 2000, while regarding not employed parents (1996–2000) and living at busy road (1991–2000) did not, but even deteriorated. 6-year old children with low parental educational level, who lived at the time of re-unification, were often under damp housing conditions and with single oven heating at homes. Nevertheless, this social inequality has almost vanished ten years later. In contrast, we found an increasing gap between low and high parental educational level with respect to the proportion of parents who were not employed (22%: 4% gain), or lived under cramped housing conditions (22%: 37% reduction), or close to a busy road (7% gain: 2% reduction).

**Conclusion:**

The social inequalities which already existed under the socialist system in East Germany persisted in the system of social market economy between 1991 and 2000. 6-year-old children from families with the lowest social status were living under the worst domestic conditions (e. g. living at busy road, having damp housing conditions, single oven heating and small living space) and for some conditions (e. g. living at busy road and having small living space) the gap betweenlow and high social status was even bigger in 2000 than in 1991.

## Background

At the turn of the 21st century, social inequalities in health continue to be a key public health problem in advanced societies, including European countries [1]. With regard to mortality, mean difference in life expectancy between those at the top and at the bottom of a society, as defined by education, income and employment status, are anywhere from 4 to 10 years [1]. In recognition of the importance of social inequalities in health, the World Health Organization (WHO) has set a special goal of reducing these inequalities in its global program 'Health for all in the Year 2000' [2,3]. Improved living conditions have contributed to better health and a decrease in death rates across all classes in developed countries [4]. Ecological studies suggest that living areas which are highly contaminated with air pollutants are over-proportionately inhabited by people of lower socio-economic status [5]. Studies done immediately after the German re-unification [5,6] demonstrated that social inequalities in health-related living conditions already existed under the social system in East Germany. In both parts of Germany children of the lower class were more likely to live at busy streets and were therefore exposed to a greater degree to traffic-related air pollution and a higher noise level [5]. Dwellings of people of lower social class were more likely to be located near industrial areas, and there was less room per person in the houses [5]. Gas was used more frequently for cooking and warm water supply, and finally the dwellings were more frequently heated with individual coal-burning stoves than upper class dwellings [5].

After Germany re-unified in 1990, dramatic social, economic and environmental changes happened during the first decade in East Germany, the former socialist GDR. The aim of this study was to test the hypothesis that the changes in East Germany had influenced social inequality in health-related living conditions of 6-year-old children during the ten-year period starting a few months after the German re-unification in 1990. This investigation has not been done before.

## Methods

### Study areas

The data for our study are derived from an environmental epidemiological study, organized by the Medical Institute for Environmental Hygiene Duesseldorf and the District Hygiene Institute of Magdeburg. The purpose of this study was to investigate the health outcomes of school beginners influenced by air pollution from 1991 to 2000 [7]. The study was conducted in the cities of Leipzig, Halle, Magdeburg, and several small rural towns. Leipzig is one of the largest cities (with about 492,000 inhabitants) in Saxony. The specific study area (South-West Leipzig) is characterized by old dilapidated housing close to small-scale industry. Halle is an industrialized city in Saxony-Anhalt with 238,000 inhabitants. Magdeburg, with about 236,000 inhabitants, is the capital of Saxony-Anhalt. The rural areas used as reference in the environmental study are the small towns Salzwedel, Gardelegen, Osterburg and Kloetze in the Altmark.

### Study design and data collection

Since 1991 cross-sectional investigations have been repeated every year until 2000 in East Germany. All boys and girls entering the elementary school from 1991 to 2000 and living in the geographically defined areas of East Germany were eligible to participate. A letter was mailed to the parents asking for participation of the child and for completion of a questionnaire at home. Written consent of the parents of the examined children was requested. During the ten-year period, altogether 25,864 6-year-old children (response 83%) participated in the environmental study. 99.2% of the children in the study (51.2% boys and 48.8% girls) had the German nationality. Every third year the questionnaire investigation was extended to cover the whole city area of Halle, Magdeburg and Salzwedel.

### Measures

#### Parental educational level

The main social indicator for this study is the parental educational level. Parental respondents reported their education and the education of their current partner if applicable. These responses served as the basis for calculating the educational level of the most highly educated parent of a child. Parental educational level was classified into three categories by the highest school grade (years) completed by either the mother or the father as follows: less than 10 years = 'low'; 10 years = 'middle'; more than 10 years = 'high' [8].

#### Parental employment status

Parental employment status was classified into two categories: mother and/or father 'in paid employment' (including full-time and part-time jobs) and 'not in paid employment' (including those parents who were not in the paid labour force, i.e. the income of the child's household was not based on regular paid labour). In the following, we will shortly refer to 'not in paid employment' as 'not employed'. The data on employment of both mother and father were only available from 1996 to 2000 for this study, because the information on employment status was not asked for before 1996.

#### Living space

We have also asked for the number of persons living in the children's homes and for the dimensions of the homes. The questions were "How many people are living in the child's home?" and "How many square meters is the child's home?" Per capita living space was defined by square meter/per person. We divided per capita living space into two groups: < 20 m^2 ^('small living space') and ≥ 20 m^2^.

#### Health-related living conditions

The questionnaire included living conditions which were considered as relevant for children's' health. Parents were asked about the type of heating in the home, whether the house could be described as damp and how far the next street with heavy traffic was. Here, 'damp housing condition', 'single oven heating' (single room heating with coke, gas, oil) and 'living at busy road' (distance to traffic street < 50 m) [9] are considered as being unfavourable health-related living conditions.

#### Region

The distribution of the socio-economic status and, similar, of the educational level is different between big cities and rural areas. Therefore, we introduced in the analysis of this study a variable which distinguished urban from rural area as potential confounder. The urban area included the cities Leipzig, Halle and Magdeburg; while the rural area included the small rural towns Salzwedel, Gardlegen, Kloetze and Osterburg.

### Statistical analyses

The data were analysed with the SAS statistical software, Version 8.2 [10]. The time course of variables was described by the included graphics. Cross tables were used to show relationships between social variables, regions and educational levels; a chi-square test was used to test associations. Multiple logistic regression analyses were conducted to evaluate the influence of educational level, region and time as independent variables on the health-related living conditions as dependent variables. As to determine an increase or decrease of the difference between lower and higher social status with respect to the dependent variable, the interaction of time and educational level was modelled too:



where p is the probability for the health-related living condition present; M and L are equal 1 if the educational level is 'middle' or 'low', respectively, else 0; T (0,1,2,...,9) represents the year of observation; R is equal 1 for urban area else 0. The estimated model parameters b_i _were presented as adjusted odds ratios together with their corresponding 95% confidence intervals to indicate the impact of the specified independent variable on the particular health-related living condition adjusted for all other independent variables of the regression model. The 'high' parental educational level served as reference category for both the 'low' and 'middle' parental educational level. The influence of the urban area environment was compared to the rural environment. The odds ratio for the factor time refers to a one-year increment. The significance of the interaction term was indicated by the two significance levels p < 0.05 and p < 0.01, respectively.

## Results

### Descriptive analyses

**Figure 1:**
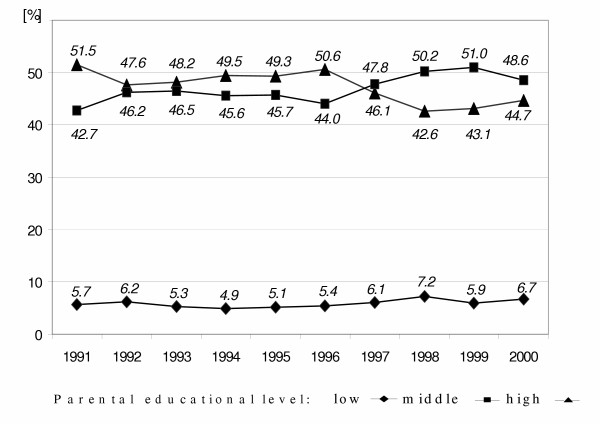
Time courses of sample distribution of parental educational level classified by the highest school grade completed by either mother or father of 6-year-old children in East Germany from 1991 to 2000 ('low': less than grade 10, 'middle': grade 10, 'high': more than grade 10);

**Figure 2:**
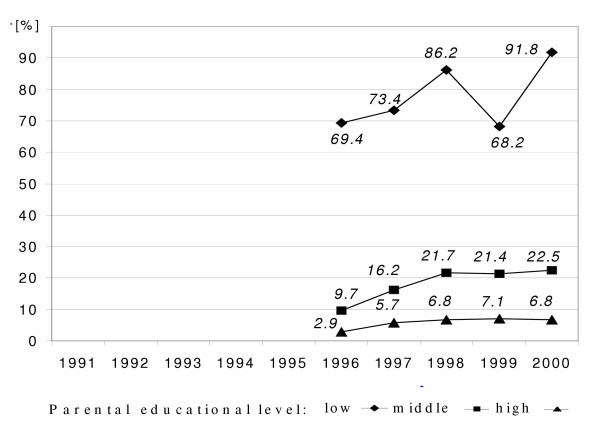
Time courses of prevalence of mother and father not in paid employment of 6-year-old children by parental educational level in East Germany from 1991 to 2000 ('low': less than grade 10, 'middle': grade 10, 'high': more than grade 10);

**Figure 3:**
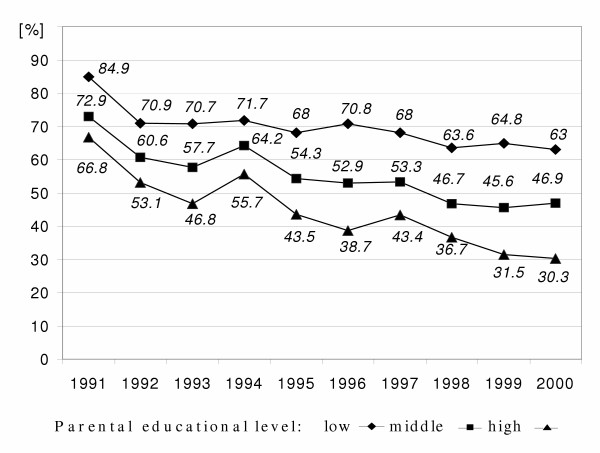
Time courses of prevalence of 6-year-old children's home with small living space (per-capita less than 20 m^2^) by parental educational level in East Germany from 1991 to 2000 ('low': less than grade 10, 'middle': grade 10, 'high': more than grade 10);

**Figure 4:**
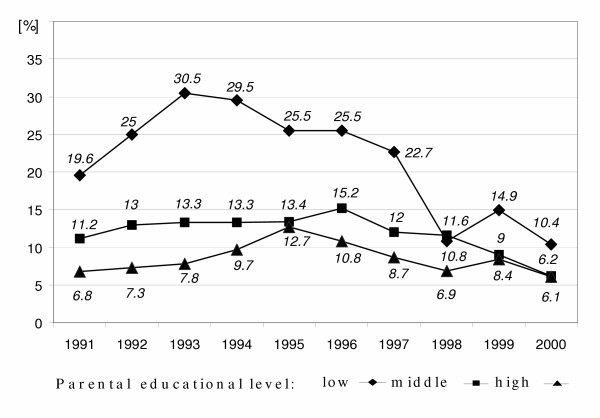
Time courses of prevalence of 6-year-old children's living under damp housing condition by parental educational level in East Germany from 1991 to 2000 ('low': less than grade 10, 'middle': grade 10, 'high': more than grade 10);

**Figure 5:**
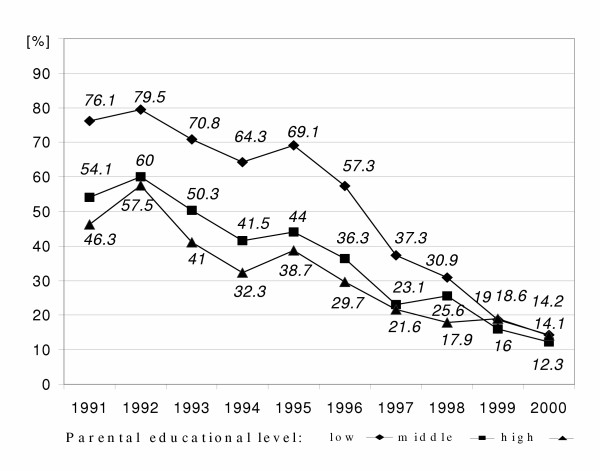
Time courses of prevalence of 6-year-old children's home with single oven heating system by parental educational level in East Germany from 1991 to 2000 ('low': less than grade 10, 'middle': grade 10, 'high': more than grade 10);

**Figure 6:**
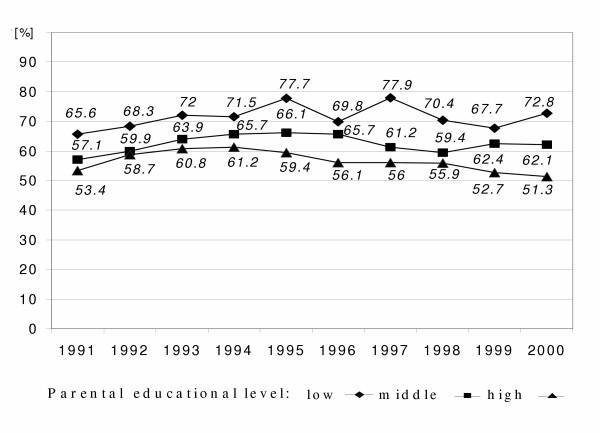
Time courses of prevalence of 6-year-old children living at busy road (distance closer than 50 m to a traffic street) by parental educational level in East Germany from 1991 to 2000 ('low': less than grade 10, 'middle': grade 10, 'high': more than grade 10).

Table 1 shows the sample size and the distribution of parental educational level by study region from 1991 to 2000. Figures 1, 2, 3, 4, 5, 6 present the time courses of the frequencies of parental social indicators and health-related living conditions of 6-year-old children in East Germany from 1991 to 2000.

**Table 1:** Distribution of parental education ('low': less than grade 10, 'middle': grade 10, 'high': more than grade 10) of 6-year-old children in East Germany (1991–2000), by study region and year

Year	Urban^a^				Rural^b^				Total(n)
	Urban (n)	Low (%)	Middle (%)	High (%)	Rural (n)	Low (%)	Middle (%)	High (%)	
1991	3125	5.2	41.4	53.4	922	7.7	47.2	45.1	4047
1992	1225	7.2	42.9	50.0	924	5.0	50.5	44.5	2149
1993	983	6.0	43.3	50.7	878	4.6	50.1	45.3	1861
1994	3814	4.9	43.4	51.8	901	5.2	54.8	40.0	4715
1995	1223	5.6	42.0	52.3	746	4.2	51.6	44.2	1969
1996	1097	5.0	41.1	53.9	688	6.0	48.6	45.5	1785
1997	2390	6.2	46.9	46.9	570	5.8	51.6	42.6	2960
1998	891	6.7	50.1	43.2	325	8.6	50.5	40.9	1216
1999	842	5.6	50.4	44.1	359	6.7	52.4	41.0	1201
2000	1837	6.8	47.6	45.6	339	6.5	53.7	39.8	2176

^*a *^Leipzig, Halle and Magdeburg; ^*b *^Salzwedel, Gardelegen, Osterburg and Kloetze.

More than 90% of children's parents in the study sample (Figure 1) received 10 or more years of school education. A complete reverse of proportions of high to middle educational level can be seen since 1997.

We found a rising rate of parents not being in paid employment at all parental educational levels (Figure 2). Despite the very high rate of not employed parents among the low educated parents in 1996, we observe a further increase of 20% until 2000 for this group.

In general, the percentage of children with homes of small living space was decreasing for all parental educational levels, but with the highest decreasing rate for the high educational level and the lowest for the low educational level (Figure 3).

The proportion of damp housing conditions had partially increased (Figure 4) until 1996 in East Germany, but thereafter, reconstruction measures of homes around that time might be responsible for the slight decline of the frequency of damp housing conditions. The difference decreased by about 10% when comparing low educational level to high educational level during the study period.

A very clear improvement of the situation regarding single oven heating systems at children's homes was found (Figure 5) and the related differences in parental educational levels extremely decreased by about 30% when comparing low educational level with high educational level over time.

A slight increase of the proportion of children living closer than 50 m to traffic street could be observed (Figure 6) for low and middle parental educational levels, whereas for high parental educational level after a slight increase during the first five years the proportion reached in 2000 nearly the same level as in 1991. The difference increased by about 10% when comparing low educational level with high educational level during the study period.

Table 2 shows a slight shift of parental education to the lower levels in rural regions compared to urban areas. Explanations for this difference between rural and urban areas could be, first, the fact that the educational level is lower in rural regions compared to larger cities or, second, the consequence of different migration after re-unification or, third, the result of a different response behaviour. Table 2 also demonstrates that the health-related living conditions were consistently better in rural areas compared to urban areas. The strongly significant differences between the three parental educational levels with respect to not employed and unfavourable health-related living conditions over the whole study period are clearly demonstrated in Table 3.

**Table 2:** Distribution of parental education, parental employment status, small living space and health-related living conditions of 6-year-old children in East Germany (1991–2000), by study region

	*Total*	*Urban^a^*		*Rural^b^*		*p-value^c^*
	*n*	*n*	*%*	*n*	*%*	*c*
Parental educational level (highest school grade):						
Low (less than grade 10)	1380	997	5.7	383	5.8	
Middle (grade 10)	11113	7730	44.4	3383	50.9	<.0001
High (more than grade 10)	11586	8700	50.0	2886	43.4	
Mother and father not in paid employment	738	599	14.6	139	10.2	<.0001
Small living space (per-capita < 20 m^2^)	13570	10133	56.9	3437	50.5	<.0001
Damp housing condition	2617	2115	12.1	502	7.5	<.0001
Single oven heating at child's home	8617	6725	40.4	1892	29.5	<.0001
Living at busy road (distance < 50 m to traffic)	14482	10812	62.0	3670	55.3	<.0001

^*a *^Leipzig, Halle and Magdeburg; ^*b *^Salzwedel, Gardelegen, Osterburg and Kloetze; ^*c *^p-value of chi-square test

**Table 3:** Distribution of parental employment status, small living space and health-related living conditions of 6-year-old children in East Germany (1991–2000), by parental educational level

	Parental educational level							
	Total	Low^a^		Middle^b^		High^c^		p-valued
		n	%	n	%	n	%	
Mother and father not in paid employment	730	157	78.5	408	17.9	165	5.64	<.0001
Small living space (per-capita < 20 m^2^)	13163	982	71.2	6514	58.6	5667	48.9	<.0001
Damp housing condition	2581	295	22.0	1306	11.9	980	8.5	<.0001
Single oven heating at child's home	8472	682	56.0	3993	38.6	3797	34.2	<.0001
Living at busy road (< 50 m to traffic)	14238	921	71.3	6785	62.3	6532	56.9	<.0001

^a ^Low: less than school grade 10, ^b ^middle: grade 10, ^c ^high: more than grade 10; ^d^p-value of chi-square test

### Logistic regression analyses

In Table 4, odds ratios, estimated by logistic regression and, therefore, mutually adjusted for the independent variables of the regression model, quantify the association between the health-related living conditions of the 6-year old children and their determinants parental educational level, time and region, respectively. Differences of change over time between the three educational levels are documented by strata-specific odds ratios, and their significance is indicated by the significance level of the respective interaction term of the regression model. The odds ratios were all significantly greater 1 for not employed, small living space, damp housing conditions, single oven heating and living at busy road when comparing low and middle educational levels to high educational level in accordance with the results in Table 3.

**Table 4:** Adjusted ^§ ^odds ratios (OR) with 95% confidence intervals (95% CI) of parental employment status and health-related living conditions of 6-year-old children in East Germany (1991–2000) for the determinants parental educational level, time and region, by means of logistic regression analysis

Independent variables	Dependent variables – OR (95%CI)				
	Mother and father not in paid employment	Small living space (per-capita < 20 m^2^)	Damp housing condition	Single oven heating at child's home	Living at busy road (< 50 m to traffic)
	N = 5406	N = 24079	N = 23778	N = 22660	N = 23662
Parental educational level high (reference)	1.00	1.00	1.00	1.00	1.00
Parental educational level middle	2.89 (1.13–7.39)	1.34 (1.22–1.47)	1.82 (1.58–2.11)	1.55 (1.41–1.70)	1.15 (1.05–1.26)
Parental educational level low	24.82 (3.90–158.10)	2.22 (1.78–2.76)	4.28 (3.38–5.41)	4.83 (3.83–6.10)	1.57 (1.27–1.94)
Time (parental educational level high)	1.16 (1.04–1.29)	0.85 (0.84–0.86)	1.00 (0.98–1.03)	0.82 (0.80–0.83)	0.98 (0.97–1.00)
Time (parental educational level middle)	1.20 (1.13–1.29)	0.89** (0.88–0.90)	0.95** (0.91–1.00)	0.78** (0.77–0.79)	1.01** (0.99–1.02)
Time (parental educational level low)	1.34 (1.09–1.71)	0.90** (0.87–0.94)	0.92** (0.88–0.96)	0.71** (0.23–0.74)	1.03* (0.90–1.18)
Region rural area^a^ (reference)	1.00	1.00	1.00	1.00	1.00
Region urban area^b^	1.55 (1.25–1.93)	1.43 (1.35–1.51)	1.78 (1.61–1.97)	1.90 (1.78–2.04)	1.35 (1.27–1.43)

§ Adjusted for all other factors included in the model and give in the Table.

^*a *^Region rural area: Salzwedel, Gardlegen, Osterburg and Kloetze; ^*b *^urban area: Leipzig, Halle and Magdeburg,

* p-value for interaction term of the regression model: p < 0.05

** p-value for interaction term of the regression model: p <0.01

The odds for not employed increased by 34% for low educational level and by 20% for middle educational level compared to 16% for high educational level per year. Despite the clear difference in change over time, the interaction terms were not significant. The odds for small living space decreased by about 10% for low and middle educational levels compared to 15% for high educational level per year. The differences in change over time were significant in favour for the higher educational level. The odds for damp housing conditions slightly, but significantly decreased over time for low and middle educational levels, but not for high educational level. The prevalence of single oven heating strongly decreased for all three educational levels over time, but the low educational level showed the strongest decline of prevalence. Living at busy road slightly, but significantly increased for low and middle educational levels compared to high educational level over time.

If comparing urban areas to rural areas, the adjusted odds for not employed, small living space, damp housing conditions, single oven heating at child's home and living at busy road, respectively, were significantly higher in urban areas.

## Discussion

The present study provides new important information on the details of changing social inequality with respect to health-related living conditions of 6-year-old children in the former socialist country, East Germany, on its transition to social market economy. The data reveals that the parental educational level as a main social indicator was consistently and significantly linked to parental employment status, living space and health-related living conditions during the whole study from 1991 to 2000 (Table 3, Figure 1, 2, 3, 4, 5, 6). The time courses of the two social indicators, employment status (both parents not in paid employment) and living space (per capita living space less than 20 m^2^), respectively, developed unfavourably and less favourably, respectively, for the two lower educational levels. For the health-related living condition 'living at busy road' (distance less than 50 m), we also observed an increasing, almost doubling gap between different social status to the disadvantage of children with low educated parents. The proportions of children living under damp housing conditions or being exposed at home to single oven heating were significantly different between the educational levels at the time of re-unification, but, fortunately, nearly identical after 10 years.

The East German society of the former socialist GDR was regarded as characterized by a relatively uniform distribution of resources and living conditions. The standard of living was higher than that in other communist countries, and a comprehensive social insurance system covered medical, disability, unemployment, and other expenses [11]. Education was free and compulsory through 10th grade [11], the economic structure was similar to that in the Soviet Union [11], with state ownership and centralized control [11], income differences between social groups were relatively small, basic health care was equally accessible to all groups of the society [8]. Although living conditions and access to consumer goods in former socialist countries were much more uniform than in the West, disparities between social groups did exist, as shown by our study. The health-related variables of our study were damp housing conditions, single oven heating and living at busy road. Several large epidemiological studies have identified damp living conditions as a major risk factor for respiratory symptoms in children [12]. There are only few publications on health inequalities in GDR and it is difficult to assess whether health inequalities do have different reasons in East Germany [13]. Although equality was a major political goal in the GDR there have been health inequalities comparable to those in Western European countries [13].

Conditions after re-unification were not easy for the East German labour force as the difficulties of adjustment to the Western system caused many hardships, including unemployment [14]. By 1991, a massive decline in employment with a loss of about 3.5 million jobs (35% of the labour force) by the end of 1992; unemployment rose from almost zero at the beginning of 1990 to 15.4% of the labour force in 1992 [15]. In our study (Table 4), odds for parents not in paid employment increased in the average per year by 34% for 'low' educated parents, by 20% for 'middle' and by 16% for 'high'. Since 1989, about 1.7 million East Germans left for the West, the birth rate fell by about 60% in the period from 1989 to 1994, and the number of marriages and divorces declined sharply [16]. In 1991 (Figure 1), about 50% of the parents had an educational level of more than 10 years of school, but, after 1996, this proportion dropped to 40% and the proportion of parents with 10 years school education raised to 50%. 1997 is the first study year where children born after the German re-unification have been investigated. Although the size of the investigation areas were not changed during the time course of the study, the number of investigated children decreased from 4047 children in 1991 to 2960 in 1997. This massive decrease can only partly be explained by the 10 percent decrease in response rate (93% in 1991 and 83% in 1997), but mostly by the massive decline in birth rates and migration to other areas. 1997 is the same year when the percentage of middle educated parents outnumbered the percentage of highly educated parents for the first time. This means that the decrease in numbers due to declining birth rates and migration was steeper to children with highly educated parents than for children with middle educated parents. This observation has also been made by other groups [personal communication]. Around the year 1994, we observed an increase of 'small living space' which was caused by the different composition of the surveys between 1993 and 1995. While the survey in 1993 had a proportion of 47% of school beginners in rural areas, the survey in 1994 showed just a fraction of 19%, whereas this fraction increased again to 38% in the survey 1995 (see Table 1).

The cross-sectional design of our study could only identify associations between social status and health-related living conditions, but could not provide the evidence of a causal relation by itself. For instance, the causal chain from a low parental educational level over low income to a cheap or unfavourable (with respect to children's health) home could not be explored. Two further limitations of this study need to be discussed. First, in the analyses presented in this paper we have considered social status differences by using parental educational level (of the most highly educated parent) for classifying children. The information about household income and occupational status of parents had not been asked in this environmental study. But an expert committee in Germany also recommended using educational level as a measure for social status in epidemiological and social medical studies [17]. Education is an important determinant of individuals' work and economic circumstances, which are themselves linked to health through specific work conditions and levels of consumption [18], related to health outcomes through its influence on lifestyle behaviours (e.g. exercise, diet), problem-solving capacities and values (e.g., importance of preventive health behaviours) [19]. Education as 'year of education completed', is a core variable in the MONICA project [13]. Nevertheless, the choice of a single indicator of parents' socioeconomic status may be subject to debate [20]. The educational level paralleled factors which are considered to be characteristic of modern domestic comfort, such as central heating, and was inversely related to the degree of crowding in the home. The second limitation was that the environmental study from which the data for this study were derived focused on airway diseases and atopic manifestations and, therefore, the selection of living conditions of the 6-year old children was restricted to their relevance for these health outcomes. Other equally important health-relevant living conditions were missing in our study, such as nutrition or access to health services.

## Conclusion

The results of our repeated cross-sectional studies in East Germany are in agreement with the well known fact of a strong association between parental educational level and health-related living conditions of children. Furthermore, we observed that the domestic situations of small living space, damp housing condition and single oven heating system improved from 1991 to 2000; while parental employment status (1996–2000) and living at busy road (1991–2000) did not improve, but even deteriorated. Our basic hypothesis that the changes in East Germany had influenced social inequality was confirmed. We found decreased social inequality for single oven heating and damp housing condition; while increased social inequality was seen for parental employment status, small living space and living at busy road. These findings lead to the conclusion that the lower social class is often, but not always the looser of large socio-economic changes as happened in East Germany. The "winning" effect seems to be bound on strong general improvements, such as the replacement of single oven heating by central heating.

A further level of investigation may be to ask how these social differences in health-related living conditions modify the association between exposure to environmental pollution and health. We know little about the effect modification of social inequality on environmental hazards or about the impact of social inequality on environment-related disease. In future, a closer co-operation between environmental-epidemiological and socio-epidemiological research would be needed.

## Competing interests

The author(s) declare that they have no competing interests.

## Authors' contributions

XDP wrote the paper and performed the statistical analysis. UK coordinated the epidemiological cross-sectional studies in East Germany. UR designed the study, assisted the statistical analysis and helped to draft the manuscript. All authors reviewed the final version of the manuscript.

## Pre-publication history

The pre-publication history for this paper can be accessed here:


